# National medicines policy development, Saudi Arabia 

**DOI:** 10.2471/BLT.22.287936

**Published:** 2022-06-22

**Authors:** Khalid Almoteiry, Adel Alharf, Bandar Al Hammad, Ibrahim Aljuffali, Nahar Al-Azemi, Taghred Al-Ghaith, Shahad Alhomidi, Ahlam Alshehri, Andreas Seiter, Christopher Herbst, Elizabeth Pisani

**Affiliations:** a Saudi Health Council, Riyadh, Saudi Arabia.; b Saudi Food and Drug Authority, Riyadh, Saudi Arabia.; c Ministry of Health, Riyadh, Saudi Arabia.; d The World Bank Group, Washington, DC, United States of America.; e Policy Institute, King's College London, Virginia Woolf Building, 22 Kingsway, London WC2B 6LE, England.

## Abstract

Medicines are at the core of every health system. The World Health Organization recommends countries develop national medicines policies that guide production, procurement, prescription and provision of medicines so that people can access the medicines they need at prices they can afford, while avoiding irrational use. However, the development of such policies is rarely straightforward. We describe important components of the national medicines policy in Saudi Arabia, which was developed within a broader transformation of the health system and the economy. The new policy formalizes existing best practices, shapes emerging policies and sets a direction for future development in four main areas. First, the policy seeks to consolidate institutional roles to provide greater cohesion; second it aims to reshape procurement and prescribing habits, with a greater focus on cost containment; third, it lays out policies which focus on assuring a secure supply of good-quality medicines, including essential medicines with limited profit potential and new products. Finally, the policy supports the growth of the domestic pharmaceutical industry, including the development of human resources. Many sectors and institutions joined in the development of the medicines policy, which was underpinned by a review of the past and current pharmaceutical context in Saudi Arabia, and good practices globally. The resulting policy was built on evidence and endeavours to give clear direction to the pharmaceutical industry and implementing agencies on rules and requirements, professional norms and institutional roles. At the same time, it maintains flexibility to allow for adaptation in a rapidly evolving institutional landscape.

## Résumé

Les médicaments sont au cœur de tout système de santé. L'Organisation mondiale de la Santé recommande aux pays d'élaborer des politiques pharmaceutiques nationales qui déterminent la production, l'obtention, la prescription et la fourniture de médicaments, afin que la population puisse accéder aux traitements requis à un prix abordable sans toutefois tomber dans un usage irrationnel. Mais cette élaboration est rarement simple. Dans le présent document, nous abordons les principaux aspects de la politique pharmaceutique nationale en Arabie saoudite, développée dans le cadre d'une vaste transformation de l'économie et du système de santé. Cette nouvelle politique officialise les bonnes pratiques existantes, façonne les projets émergents et définit une orientation pour l'évolution future dans quatre domaines clés. Premièrement, elle cherche à renforcer le rôle des institutions pour améliorer la cohésion. Deuxièmement, elle vise à remanier les habitudes d'obtention et de prescription, en se focalisant sur la maîtrise des coûts. Troisièmement, elle établit des mesures destinées à assurer un approvisionnement sûr en médicaments de qualité, y compris en nouveaux produits et médicaments essentiels au potentiel de rentabilité limité. Et quatrièmement, elle soutient la croissance de l'industrie pharmaceutique dans le pays, notamment le développement des ressources humaines. De nombreux secteurs et institutions ont contribué à l'élaboration de cette politique, qui s'appuie sur une analyse du contexte pharmaceutique antérieur et actuel en Arabie saoudite ainsi que sur les bonnes pratiques mondiales. Le résultat repose sur des éléments et efforts tangibles. Il montre clairement à l'industrie pharmaceutique et aux organismes de mise en œuvre la marche à suivre en matière de règles et d'exigences, de normes professionnelles et de rôles institutionnels. Et dans le même temps, il prévoit suffisamment de flexibilité pour s'adapter à un paysage institutionnel en constante mutation.

## Resumen

Los medicamentos son el núcleo de todo el sistema sanitario. La Organización Mundial de la Salud recomienda a los países que desarrollen políticas nacionales de medicamentos que guíen la producción, la adquisición, la prescripción y el suministro de estos, de modo que las personas puedan acceder a los medicamentos que necesitan a precios que puedan pagar, evitando al mismo tiempo un uso irracional. Sin embargo, la elaboración de estas políticas no suele ser sencilla. En este documento se describen componentes importantes de la política nacional de medicamentos en Arabia Saudita, que se desarrolló en el marco de una transformación más amplia del sistema sanitario y la economía. La nueva política formaliza las mejores prácticas existentes, da forma a las políticas emergentes y establece una dirección para el desarrollo futuro en cuatro áreas principales. En primer lugar, la política busca consolidar las funciones institucionales para proporcionar una mayor cohesión; en segundo lugar, pretende remodelar los hábitos de adquisición y prescripción, centrándose más en la contención de los costes; en tercer lugar, establece políticas que se centran en asegurar el suministro de medicamentos de buena calidad, incluidos los productos nuevos y los medicamentos esenciales con un potencial de beneficio limitado. Por último, la política apoya el crecimiento de la industria farmacéutica nacional, incluido el desarrollo de los recursos humanos. Varios sectores e instituciones participaron en la elaboración de la política de medicamentos, que se sustentó en una revisión del contexto farmacéutico pasado y actual en Arabia Saudita, y de las buenas prácticas a nivel mundial. La política resultante se creó a partir de pruebas y se esfuerza por dar una orientación clara a la industria farmacéutica y a los organismos de ejecución sobre las reglas y requisitos, las normas profesionales y las funciones institucionales. Asimismo, mantiene la flexibilidad para permitir la adaptación en un panorama institucional que evoluciona muy rápido.

## ملخص

الأدوية هي الأساس في كل نظام صحي. توصي منظمة الصحة العالمية الدول بتطوير سياسات الأدوية الوطنية التي توجه إنتاج الأدوية وشرائها ووصفها وتوفيرها، حتى يتمكن الناس من الحصول على الأدوية التي يحتاجون إليها بأسعار في متناولهم، مع تجنب الاستخدام غير الرشيد. ومع ذلك، فإن تطوير مثل هذه السياسات نادرًا ما يكون واضح المعالم. نحن نصف المكونات المهمة لسياسة الأدوية الوطنية في المملكة العربية السعودية، والتي تم تطويرها ضمن تحول أوسع للنظام الصحي والاقتصاد. تضفي السياسة الجديدة الطابع الرسمي على أفضل الممارسات الحالية، وتشكل السياسات الناشئة، وتحدد اتجاه التنمية المستقبلية في أربعة مجالات رئيسية. أولاً، تسعى السياسة إلى تعزيز الأدوار المؤسسية لتوفير قدر أكبر من التماسك؛ وثانيًا، وتهدف إلى إعادة تشكيل عادات الشراء والوصف، مع التركيز بشكل أكبر على احتواء التكلفة؛ وثالثًا، تضع السياسات التي تركز على ضمان الإمداد الآمن بالأدوية عالية الجودة، بما في ذلك الأدوية الأساسية ذات الإمكانات المحدودة للربح والمنتجات الجديدة. وأخيرًا، تدعم السياسة نمو القطاع الدوائي المحلي، بما في ذلك تنمية الموارد البشرية. انضمت العديد من القطاعات والمؤسسات إلى تطوير سياسة الأدوية، والتي استندت إلى مراجعة السياق الدوائي السابق والحالي في المملكة العربية السعودية، والممارسات الجيدة على الصعيد العالمي. تم بناء السياسة الناتجة على الأدلة والمحاولات لإعطاء توجيه واضح للقطاع الدوائي والوكالات المنفذة بشأن القواعد والمتطلبات، والمعايير المهنية والأدوار المؤسسية. في الوقت ذاته، تحافظ على المرونة للسماح بالتكيف في مشهد مؤسسي سريع التطور.

## 摘要

药品是每个医疗体系的核心。世界卫生组织建议各国制定国家药物政策，以指导药物的生产、采购、处方和供应，使人们能够以负担得起的价格获得所需的药品，同时避免药品的不合理使用。然而，此类政策的制定并不容易。我们描述了沙特阿拉伯国家药物政策的重要组成部分，该政策是在医疗体系和经济进行大转型的时期制定的。新政策正式确定了现有的最佳实践，勾勒了新兴产业政策，并通过四个主要方面指明未来发展的方向。首先，该政策旨在巩固机构角色，进而提高行业凝聚力；其次，它旨在重塑采购和处方习惯，更加注重成本控制；第三，它制定了侧重于确保优质药品安全供应的政策，包括利润空间不大的基本药品和新药品。最后，政策支持国内制药行业的发展，包括人力资源的发展。许多部门和机构参与了药物政策的制定，通过审查沙特阿拉伯制药行业的过去与现状以及全球良好实践来提升政策制定水平。由此制定的政策建立在证据的基础上，为制药行业和执行机构在药物的规则和要求、专业规范和制度方面提供明确的方向。同时，它还保持了灵活性，以适应快速发展的制度环境。

## Резюме

Лекарственные средства лежат в основе любой системы здравоохранения. Всемирная организация здравоохранения рекомендует странам разработать национальную политику в отношении лекарственных средств, которая регулировала бы производство, приобретение, назначение и предоставление лекарственных средств, чтобы люди могли получать доступ к необходимым им лекарствам по доступным ценам, избегая при этом нерационального использования. Однако разработка такой политики нередко вызывает затруднения. Авторы описывают важные компоненты национальной политики в области лекарственных средств в Саудовской Аравии, которая была разработана в рамках более широкой трансформации системы здравоохранения и экономики. Новая политика формализует существующую передовую практику, формирует новую политику и определяет направление будущего развития в четырех основных областях. Во-первых, политика направлена на консолидацию институциональных функций для обеспечения большей сплоченности. Во-вторых, она направлена на изменение практик в области приобретения и назначения лекарств при особом внимании к сдерживанию расходов. В-третьих, в ней излагаются политики, направленные на обеспечение бесперебойных поставок качественных лекарственных средств, в том числе основных лекарств с ограниченным потенциалом прибыли, а также новых препаратов. Наконец, политика поддерживает рост отечественной фармацевтической промышленности, включая формирование кадрового резерва. Многие секторы и учреждения присоединились к разработке политики в отношении лекарственных средств, которая основывалась на обзоре прошлого и текущего фармацевтического контекста в Саудовской Аравии, а также на передовом мировом опыте. Соответствующая политика была построена на фактических данных и попытках дать четкие указания фармацевтической промышленности и учреждениям-исполнителям в отношении правил и требований, профессиональных норм и институциональных функций. В то же время она сохраняет гибкость, позволяющую адаптироваться к быстро меняющемуся институциональному ландшафту.

## Introduction

Pharmaceuticals – medicines, vaccines and diagnostic tests – and medical equipment are at the centre of any health system, and are central to good clinical practice. The World Health Organization (WHO) recommends that countries develop national medicines policies that lay out the medium- and long-term aims in relation to pharmaceuticals and that provide direction to public and private actors on both the demand and the supply side of the market.^1^

The process of developing such a policy is rarely straightforward since it never occurs in a vacuum and must take existing policies, practices and contexts as its starting point. Individuals and institutions involved in developing the policy must consider the disparate, and sometimes conflicting, interests of many different groups of actors.^2^ For example, the interests of public consumers may be set against those of private suppliers; the ambitions of the industrial sector may clash with health-sector goals. In this paper, we report the key features of the national medicines policy of Saudi Arabia, which has been newly revised at a time of rapid institutional change. We describe the context in which the policy evolved, outline its priority goals and briefly discuss the policy choices made.

## Before the policy

The 2002 Health Law in Saudi Arabia aimed to provide comprehensive health care for all people in a fair and accessible manner.^3^ In 2016, the country announced a national transformation programme with the objective of reforming the economy, reducing dependence on the oil sector and boosting national resilience against global economic changes by 2030. The health ministry subsequently published a Health Sector Transformation Strategy, while the World Bank reviewed health-sector expenditure.^4^^,^^5^ These documents describe a system that was deeply fragmented; public sector health services were provided by the ministries of health, defence and interior, as well as the National Guard and autonomous specialist hospitals, while there was also extensive private provision of health services. Services were skewed towards tertiary care, with underinvestment in primary care and prevention, especially for noncommunicable diseases. The transformation strategy characterized the existing health sector as one of low productivity which focused more on the needs of institutions than patients. The report stressed that “The health system also needs to support the containment of public expenditure ... to address the risk of long term reductions in the price of crude oil and the impact that will have on public revenues.”^4^

To address these challenges, Saudi Arabian government has begun to restructure the health sector by vertically integrating primary and higher-level services, introducing a single agency that pays for all public health services and pooling procurement of medicines and equipment. At the same time, in an effort to reduce dependence on oil revenues, the government is promoting investment in other strategic industries, including pharmaceutical manufacturing.

## Evolution of the policy

**Fig. 1:**
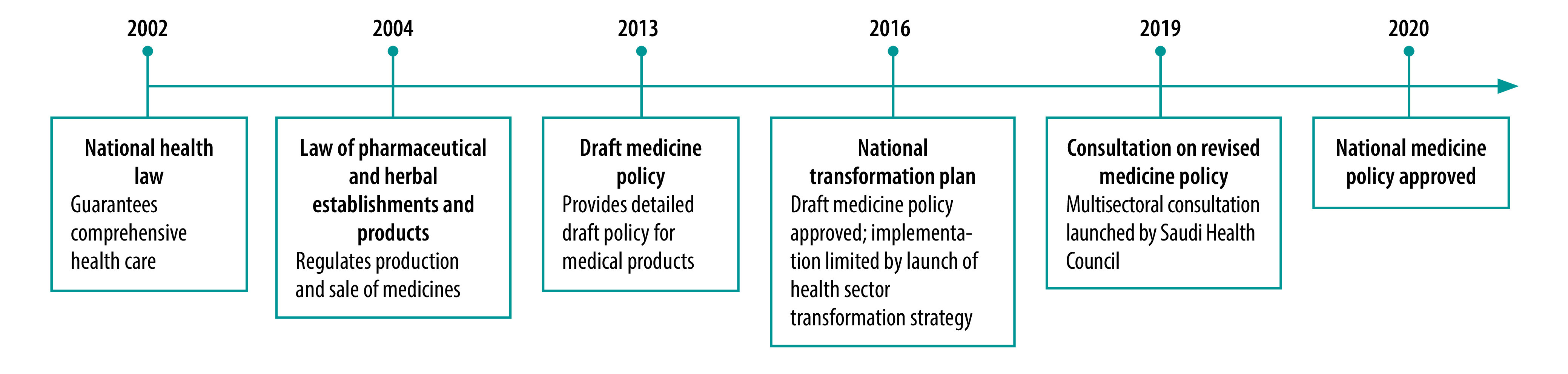
Timeline of development of the Saudi Arabian national medicines policy, 2020

Because all these changes potentially interact with the selection, procurement and use of medicines, in 2019, Saudi Arabia began to revise its national medicines policy which had its roots in laws going back 2 decades (Fig. 1).

The new policy reiterates existing policies or formalizes existing best practices where they remain appropriate. It also contains new or modified provisions in areas in which reforms are ongoing and sets goals for policies that may be needed as reforms evolve. The aim was a policy that was both sufficiently precise to provide clear guidance and sufficiently flexible to encompass the ongoing reforms and developments in the health sector.

Following the WHO guidance on developing national medicines policies, the Saudi Health Council, which coordinates policy in the health sector, first commissioned a review of existing policies and practices in the pharmaceutical sector in the country. The areas covered were: regulatory and institutional arrangements; medicine selection; measures to promote appropriate use of medicines; pricing; financing and reimbursement; procurement and supply chain policies; industrial policy; pharmaceutical security; data systems; and human resources. In addition to examining current practices in the country, the review also described policy alternatives in each of those areas and provided evidence of the effects of such alternatives from other countries and settings.^6^

The findings of this review were discussed in several national workshops, involving representatives from different national health-sector institutions, including the health ministry, the Saudi Food and Drug Authority and the National Unified Procurement Co., as well as international and regional technical organizations and academia. Differences of opinion that arose in these meetings were most often because of misunderstandings resulting from the failure of different sectors to keep one another informed of changes in policy and practice at a time of rapid change. Box 1 and Box 2 give examples of areas of policy which required particular negotiation. Where necessary, the Saudi Health Council convened additional meetings between key actors to gather more information before drafting a revised national medicines policy, which was eventually agreed by consensus. The draft policy then went through the national approval process, including consideration by the Council of Ministers and Royal Assent. The Saudi Health Council published the policy review in English together with an English translation of the policy itself,^6^ which was approved in 2020 (available in the data repository together with supplementary information on the implementation status of each policy element as of May 2022).^12^

Box 1 **Finding an acceptable path to lowering costs**

Several ministries and specialist hospitals provide health services to the Saudi population. Many are well resourced and powerful entities. At the time the medicine policy consultation was launched, each institution (and sometimes each department within an institution) developed its own formulary and procured its own medicines, often in relatively small quantities. Thus, hospital formularies were shaped by doctors’ preference for branded products, even when generic products were available, and their habit of prescribing by brand rather than using the generic or international nonproprietary name of the molecule.^5^^,^^7^ These factors combined to produce unnecessarily high spending on medicines by public institutions.

The data review that underpinned policy discussions provided strong evidence that consolidated purchasing can drive down prices, while generic prescribing and procurement practices are effective cost-containment measures (a conclusion since echoed in the World Health Organization’s guideline on country pharmaceutical pricing).^8^ An early draft of the policy recommendations mandated prescribing by molecule name, as well as the use of a single national formulary. However, during discussions about the medicines policy, representatives from some government medical institutions expressed the view that this mandate unreasonably constrained physicians’ autonomy, an opinion also described in academic research.^9^^,^^10^ These institutions were similarly resistant to a policy that would require all public sector institutions to procure medicines through the National Unified Procurement Co., which had previously only procured on behalf of the health ministry.

In the final policy, all parties arrived at a compromise. The policy includes measures to encourage prescribing using international nonproprietary names, but does not mandate such prescribing in any sector. The policy mandates the development of a national medicines reimbursement list, but describes its function only as providing guidance. Simultaneously, however, it was decided that the likely cost-efficiencies of consolidated procurement outweighed the desire of individual institutions to continue to buy medicines independently. The policy therefore mandates that all public sector institutions buy their medicines through the National Unified Procurement Co. The company does not publish absolute volumes, but has reported to the Saudi Health Council that 46% by volume of the medicines they procure are generic or biosimilar products (compared with just 24% of Saudi Arabian market volume before the reforms). The policy thus has the effect of reducing the selection of branded medicines available for doctors to prescribe to patients in any public sector institution.

Box 2 **Access to innovation**

Medicine manufacturers who wish to sell their products in Saudi Arabia have always needed approval from the Saudi Food and Drug Authority before doing so. Some companies were reluctant to follow this process because the procedure was lengthy; for example, requiring certification of product prices by the Saudi Arabian embassy in 30 reference countries.^11^ A decade ago, in deciding their market entry strategy, multinational companies waited an average of 17 months between first launching a new medicine anywhere in the world and applying for market authorization in Saudi Arabia (compared with a lag of 2 weeks to apply for approval in Canada).^11^

One result was that hospitals, which maintained their own formularies and procured on their own account (Box 1), would use waivers to import new products that were not yet authorized by the Saudi Food and Drug Authority. By the time of the discussions on the medicines policy, the Saudi Food and Drug Authority had already implemented steps that greatly reduced approval times, but the use of waivers continued. In discussion between various institutions, a pragmatic solution was found. The medicines policy clearly mandates that all medicines provided to patients in Saudi Arabia must be approved by the Saudi Food and Drug Authority, thus reducing irregular imports of unregulated products by individual institutions. However in implementing the policy, it was agreed that the National Unified Procurement Co. may import medicines that have been approved by other stringent regulatory authorities as long as the manufacturer undertakes to seek Saudi Food and Drug Authority regulatory authorization within 6 months of the importation date.

The remainder of this paper focuses on challenges and medicines policies in four areas that most closely intersect with the broader areas for transformation identified in the Health Sector Transformation Strategy and the National Transformation Plan, namely: institutional cohesion; cost containment; coverage of the spectrum of health needs; and development of domestic pharmaceutical production.

## Features of the policy 

### Institutional cohesion

As the health ministry noted in 2016, “The current Saudi health system is significantly fragmented, leaving various regulatory bodies with restricted, often overlapped, jurisdiction and authority.”^13^ In the area of medicines, the autonomous Saudi Food and Drug Authority has long been the main regulator, overseeing access to the Saudi pharmaceutical market and ensuring that products marketed are safe, effective and fairly promoted. The health ministry, for its part, regulates and licenses service providers, including retail pharmacies. Other institutions are involved in overseeing the training and conduct of pharmacists, issuing permits to industry and regulating trade. The institution that sets regulations may differ from the one that enforces them. It has been noted that, “The back and forth between many sectors can be complex, lengthy, and bureaucratic.”^14^ The reform process provided an opportunity to define more clearly the roles and responsibilities of each institution. The national medicines policy explicitly seeks to create a cohesive framework which increases synergy and reduces duplication of effort. Box 3, adapted from the policy, describes the lead responsibilities of nine different institutions or groups of actors. In addition, the policy mandates the sharing of data needed to implement and monitor the national medicines policy.

Box 3 **Primary roles of institutions, as defined in the national medicines policy and other regulations, Saudi Arabia, 2020**

***Saudi Health Council***

- Oversee the development of the national medicines policy, in consultation with all concerned sectors. Ensure the timely adoption of the policy.
- Plan and mandate cross-sectoral data contribution that will allow for the efficient monitoring and revision of the policy.
- Review data analysis and revise policy as necessary, in consultation with all concerned institutions, on a predefined schedule.
- Lead cross-sectoral consultation to define emerging policy needs and questions.
- Collate and analyse medicines data to guide the monitoring and revision of the medicines policy.

***Saudi Food and Drug Authority***

- Organize and manage the processes of registration, renewal and variations of pharmaceutical preparations (human, veterinary and herbal).
- License the processes of manufacturing, importing, exporting, distributing, promoting and advertising of medicines.
- Inspect pharmaceutical manufacturing sites.
- Assess the safety, efficacy and quality of pharmaceutical preparations, and issue marketing authorization.
- Take responsibility for pharmaceutical pricing, pricing review and evaluation of economic and clinical comparison studies in treatment groups.
- Monitor narcotic drugs, psychotropic substances and controlled preparations and ensure compliance with relevant regulations and procedures.
- Develop and manage policies, regulations and guidelines for pharmaceutical preparations.
- Conduct postmarketing surveillance of pharmaceuticals and monitoring of adverse drug reactions (pharmacovigilance).
- Secure pharmaceutical product supply in the Saudi Arabian market and monitor and address supply shortages.
- Track medicines throughout the supply chain using an electronic track and trace system to reduce fraud and ensure their safety and availability.

***Health ministry***

- Oversee the development of clinical guidelines in all major therapeutic areas.
- Develop, monitor and enforce regulations to promote cost-efficient prescribing and dispensing, including the use of international nonproprietary names.

***Center for Health Technology Assessment***

- Undertake health technology assessment of new technologies to inform decision-making on reimbursement for public and private payers.
- Establish and revise guidelines and national standards for health technology assessment methods.
- Establish guidelines for managed entry agreement between payers and manufacturers.

***National Unified Procurement Co.***

- Plan demand for the public sector.
- Procure medicines for the public sector, in line with policies on local products.
- Manage the public sector supply chain.
- Assure a sustainable supplier base for low-profit medicines.
- Maintain framework contracts for procurement and distribution of medicines in emergencies and disasters.
- Maintain the national stockpile of medicines.

***Public payer***

- Develop regulations to promote cost-efficient prescribing for reimbursement, including prescription using the generic name of a medicine rather than the brand name.
- Negotiate reimbursement prices for high-value medicines.

***Private payers***

- Develop medicine reimbursement lists based on guidance and governance by the Council of Cooperative Health Insurance.

***Academia, Saudi Commission for Health Specialties***

- Take responsibility for curriculum development, workforce licensing, training and continued education and professional development.

***National Risk Unit***

- Ensure that lead organizations integrate disaster preparedness into their respective roles.
- Coordinate the work of the lead organizations in the event of a disaster, including maintaining a rapid data exchange platform and command centre.
- Lead public communication related to medicines in the event of a disaster.

Technical data systems, such as those that track medicines through the supply chain, are advancing as scheduled. However, further work is needed to instil norms of data sharing between institutions and regularize the analysis and use of those data in monitoring and adjusting the implementation of the overall policy.

### Cost containment

In 2019, at the start of the health sector reforms, spending on medicines was estimated at 8.2 billion United States dollars (US$), over a fifth of all health spending. Estimates of the retail sales direct to patients in Saudi Arabia were 43% (US$ 3.6 billion) of medicines in terms of value and 59% (2.0 billion of 3.4 billion units) in terms of volume in early 2019.^15^ This level of out-of-pocket spending was considered high in a country which aspires to provide health coverage to all residents. At the same time, many of the medicines that were provided free may have been wasted because patients often visited more than one provider and cross-checking patient records was impossible in the fragmented health system.^16^^,^^17^ Additional inefficiencies included: high proportions of relatively expensive originator brands; high levels of polypharmacy and irrational medicine use; and the lack of any system for consolidating procurement to achieve greater bargaining power. For example, patented medicines were estimated to account for 63% (US$ 4.1 billion/6.5 billion) of prescription medicines consumed in Saudi Arabia, far higher than most countries with similar income levels.^18^

Table 1 outlines the medicines situation in Saudi Arabia before the health reforms, and the related policy responses and outcomes in the area of cost containment for medicines.

**Table 1:** Policies to contain medicine costs, Saudi Arabian medicines policy, 2020

Prereform situation	Policy response	Outcomes
Retail price capped by the Saudi Food and Drug Authority	Unchanged	Retail prices are low by regional standards
Essential medicines list existed based on international nonproprietary names but the national formulary was not consistently used across the public sector	Develop a national medicine reimbursement list for public sector based on transparent health technology assessment	Not yet implemented. May meet resistance from pharmaceutical industry and medical professions
Polypharmacy and irrational prescription were common. Clinical practice guidelines were limited and did not always consider cost–effectiveness	Develop further clinical practice guidelines, including recommendations for cost-effective medicines	Three new guidelines published since 2020
	Share electronic health records between providers to reduce duplicate prescribing	Not yet implemented
	Consider co-payments to discourage over-consumption	Mentioned in the policy as a potential future direction
Doctors often prescribed by brand	Develop programmes to incentivize prescribing with international nonproprietary names	Not yet widely implemented; may meet resistance from doctors, pharmacists and industry
	Include education in value-based prescribing and dispensing in the national curricula and conversion courses for expatriate clinicians and pharmacists	Not yet implemented
Each institution procured medicines independently	Mandate public sector procurement through the national procurement agency	Implemented; medicine prices have fallen

### Secure supply

Saudi Arabia has complex pharmaceutical needs. Epidemiologically, the country faces problems common to other high-income countries, including an increase in chronic diseases related to an ageing and largely sedentary society. Rare genetic conditions are more prevalent than in other societies of similar income level.^19^ Furthermore, infectious diseases remain a challenge because of a labour market highly dependent on immigrants from low- and middle-income countries and the hosting of millions of pilgrims from lower-income countries. Saudi Arabia must therefore manage the challenges associated with rapid access to innovative (and often expensive) biological and targeted therapies, while simultaneously securing a supply of essential medicines that offer low profit margins for producers.

Broadly speaking, Saudi Arabian citizens and residents have good access to most essential medicines; most people can get these medicines free in the public sector, although many people opt to buy medicines privately for convenience. Before the health reforms began, thorough but relatively lengthy regulatory review combined with stringent price controls sometimes impeded rapid access to innovative therapies (Box 2).^11^

The supply of low-profit medicines, on the other hand, was sometimes limited because suppliers lacked incentives to provide these medicines because orders were small as a result of the fragmented procurement landscape. In addition, regulatory and bureaucratic restrictions on procurement and importation sometimes inhibited rapid procurement in times of national emergency or during other unexpected increases in demand.

Table 2 describes the policies that have been adopted in response to these supply challenges.

**Table 2:** Policies to assure the supply of medicines, Saudi Arabian medicines policy, 2020

Prereform situation	Policy response	Outcomes
Stringent review procedures and global price benchmarking slowed entry of new products into the market	Review procedures by Saudi Food and Drug Authority to reduce duplication of effort	Policy formalized and reforms already underway; approval time greatly reduced
	Manage entry agreements for new products	Implemented; new therapies introduced more affordably and rapidly
	Develop framework procurement contracts based on emergency medicine formularies that allow for rapid response purchasing when necessary	Progress towards full implementation helped by clearly delineated role of National Risk Unit (Box 1)
Fractured demand discouraged market entry	Expand the national procurement agency to cover all public sector medicines procurement	Implemented; consolidated demand attracts suppliers, including of lower-priced medicines
	Implement a multiple winner system (more than one supplier selected per tender) to protect against supply failures	Implemented
Fragmented system impeded demand planning for medicines and stock control, creating a risk of shortages in the public system	Flag likely stock-outs through real-time monitoring of data from a national end-to-end distribution tracking system. Implement mandatory reporting of expected production shortfalls by registered manufacturers or their locally authorized representatives	Implementation ongoing
	Maintain a stockpile of essential medicines	Implemented through National Unified Procurement Co.

### Local industry promotion

When the National Transformation Program was published in 2016, almost all originator brand medicines were imported, as were 68% (US$ 5.12 billion /7.47 billion) by value of all other medicines. In its effort to reduce dependence on oil revenues, the National Transformation Program designated the pharmaceutical and biotechnology clusters as priority areas for investment and growth. The motivation for this industrial policy is largely economic rather than health-related, and the principal policy instruments are also economic. For example, domestic pharmaceutical and biotechnology companies have been provided with interest-free investment capital as well as subsidized access to land and utilities to encourage investment in the sector.^18^

The Health Sector Transformation Strategy noted, “When other government departments in the Kingdom develop major policy initiatives, the health and healthcare implications of their actions are not always at the front of their minds.”^4^ The National Medicines Policy must explicitly consider the implications of an industrial policy which specifically targets the pharmaceutical sector.

While the long-term aspiration is to produce research-based medicines, reviews of the sector recognize that the necessary skills base is not yet well developed in Saudi Arabia.^20^ It is likely that in the short term the expansion of pharmaceutical manufacturing will involve an increase in the production of generic medicines. The 27 domestic pharmaceutical manufacturers registered in Saudi Arabia currently concentrate on the production of generic medicines. In 2019, these manufacturers accounted for 15% (1.2/8.2 billion US$) to 18% (1.5/8.2 billion US$) of the national market; achievement of the National Industrial Development and Logistics Program would see this rise to 40%.^21^ Domestic production will likely displace generic medicines currently imported from high-volume, low-cost producers. Start-up costs and fewer economies of scale will likely push the price of these medicines higher than those of imported equivalents in the early years. This situation means that the National Unified Procurement Co. will pay more to procure these local products for the public sector, potentially using up other parts of the health budget. Since production capacity takes time to develop, the effects of potential price increases have not yet been seen. However, the medicines policy includes provision for as yet unspecified economic measures that protect public health budgets against price implications associated with procurement of domestically produced medicines.

Table 3 summarizes the prereform situation of the local pharmaceutical sector and policy responses to support its development. 

**Table 3:** Policies to support development of the domestic pharmaceutical sector, Saudi Arabian medicines policy, 2020

Prereform situation	Policy response	Outcomes
Some tenders were restricted to domestic producers (not formalized)	Provide formal guidance on product authorization and procurement that specifically considers local content	Ongoing; facilitated by centralized public sector procurement
Skills base in industrial pharmacy or pharmaceutical research was limited	Reform pharmacy curriculum, training programmes and other human resource development efforts to ensure needs of an emerging pharmacy sector are adequately met	Not yet implemented; requires close coordination with industry, academia, education ministry and professional bodies
Generics were imported from low-cost producers	Implement fiscal measures to protect public health budgets from substitution with more expensive local products	Measures to be further specified if price rises occur

## Outcomes and lessons learnt

No formal evaluation of the implementation of the medicines policy has yet been conducted, so we are unable to describe measured outcomes. However, the process of developing a national medicines policy has produced a result beyond the policy itself. The contextual analysis that informed the policy development process brought together a wide and disparate body of information generated by Saudi and international scholars, pharmaceutical market analysts and international technical organizations. For various areas of the medicines policy, the analysis summarized the experience of other countries, as well as the existing situation in Saudi Arabia. This information highlighted some of the vulnerabilities in the national system, for example: limited use of cost-efficient generic and biosimilar medicines; inefficient procurement; and wasteful polypharmacy. The analysis also showed some unusual features of the local market, such as the simultaneous need for medicines commonly required by high-income countries with a high burden of noncommunicable diseases and for medicines needed to prevent or treat communicable diseases more prevalent in low-income settings. The review provided strong evidence against which alternative policy choices could be discussed and evaluated, emphasizing, as WHO and others have done, the importance of the national context in driving policy choices.^1^

The workshops held by the Saudi Health Council added an important element to the desk analysis. As noted in a recently published analysis of policy-making in Saudi Arabia, there is “divergence within the reform agenda, whereby ministries, institutions and agencies are tasked with implementing reforms which conflict with one another.”^22^ In developing the medicines policy, representatives of many different institutions with knowledge of the pharmaceutical sector came together to challenge or enrich findings in the light of their experience and to provide different perspectives on potential policy choices. Besides strengthening the network of interested individuals and institutions, and facilitating further communication between them, the process highlighted some of the potential mismatches between the policy goals of different sectors. For example, industrial policies promoting domestic pharmaceutical production might in the short term increase costs to the health system, a dynamic likely to be faced by many other countries as they consider increasing local production of medicines.^23^ In drafting the policy, a provision was therefore included that committed the government to providing compensatory finance for medicines procurement, if necessary. This option may not be available to the many lower-income countries whose politicians are currently pushing for more domestic pharmaceutical manufacturing.^24^

The inclusive process also underlined the fact that different actors have different priorities; the more fragmented the health-care landscape, the more competing priorities there are likely to be. For example, doctors accustomed to directing procurement decisions for an individual hospital may wish to retain the autonomy to prescribe familiar medicines and brands. The Saudi Arabian government is hoping to persuade multinational pharmaceutical firms to invest more in medicine research and production in the country. These firms (and their local distributors) also support prescribing using brand names.^25^ Those bodies overseeing national health budgets, on the other hand, favour imposing more limited formularies including unbranded generic products. By discussing policy development within a workshop environment, the Saudi Health Council attempted to balance these interests, as illustrated in Box 1 and Box 2. In countries with more confrontational political systems, such tensions have undermined the implementation of a national medicines policy.^2^ However, as noted in a review of case studies in countries as diverse as Australia, North Macedonia, South Africa and Sri Lanka, even in more highly contested settings, dialogue between interested parties increases the likelihood of success.^2^

In the case of Saudi Arabia, the product of this dialogue is a deliberately high-level policy which aims to ensure that citizens and residents of, and visitors to, Saudi Arabia have access to a continuous supply of appropriate, safe and effective medicines which deliver the greatest amount of health for the lowest sustainable cost. This policy grew out of previous work and formalizes existing policies and practices for which detailed operational guidance often already exists and which are judged to be working well. In addition, it provides guidance for the further development of policies that are currently partially implemented. Finally, the policy provides direction on policies that are expected to become more necessary as health-sector reforms progress.

While the earlier Saudi Arabian medicines policy was more narrowly focused on regulatory issues, the present policy addresses the interests of the broader health sector and includes the interests of industry and national security. This policy was made possible by health-sector reforms and the wider national transformation agenda. Realization of the policy supports the assertion that policy development is most likely to succeed at times of dramatic institutional change.^2^ Health-system reforms of the scale of those taking place in Saudi Arabia are unusual in most high-income countries that provide health care to all citizens. Many low- and middle-income countries are however currently reforming their health systems to try and provide universal health coverage; they will be familiar with some of the challenges faced by Saudi Arabia, in particular issues of cost containment and institutional fragmentation.^26^

As the example given in Box 2 shows, and as researchers in other settings have also found,^27^ appropriate adjustment and flexible implementation may be needed, either to increase the feasibility of implementation or to maintain the support and participation of important institutions. The Saudi Arabian medicines policy mandates monitoring of progress in implementation, under the coordination of the Saudi Health Council. This process should allow the Council, together with the Saudi Food and Drug Authority and other institutions that contributed to the development of the policy, to revise the implementation of elements of the policy over time so that the main policy goal, that is, sustained and sustainable access to appropriate and effective medicine for patients in Saudi Arabia, is consistently achieved.
